# List of Assistant Surgeons Found Qualified for Promotion, and Candidates Qualified for Admission into the Navy of the U. S. as Assistant Surgeons

**Published:** 1845-01

**Authors:** 


*Of the date of October and December,* 1839.—James B. Gould, Charles H. Wheelright and John H. Wright.

Of the candidates examined for admission into the Navy as Assistant Surgeons, sixteen were found qualified in the following order of relative merit:

1. Bernard Henry, jr.; 2. Robert T. Maccon; 3. William A. Harris; 4. Robert E. Wall; 5. Washington Sherman; 6. Henry O. Mayo; 7. John Rudenstein; 8. Randolph F. Mason; 9. Philip Lansdale; 10. P. Benson D. Lany; 11. Alexander J. Rice; 12. S. Allen Paddock; 13. John A. Petit; 14. Thomas B. Steele; 15. J. F. Harrison; 16. A. N. Bell.—*Ibid.*

